# Plant stem cell extract from *Coffea canephora* shows antioxidant, anti-inflammatory, and skin regenerative properties mediated by suppression of nuclear factor-κB

**DOI:** 10.1590/1414-431X2023e12849

**Published:** 2023-06-30

**Authors:** M. Guidoni, A.D. de Sousa, V.P.M. Aragão, M.V. Toledo e Silva, T. Barth, W.R. Clarindo, D.C. Endringer, R. Scherer, M. Fronza

**Affiliations:** 1 Programa de Pós-Graduação em Ciências Farmacêuticas, Laboratório de Produtos Naturais, Universidade Vila Velha, Vila Velha, ES, Brasil; 2 Instituto Capixaba de Ciências e Administração, Programa de Desenvolvimento Científico e Tecnológico Regional, Vila Velha, ES, Brasil; 3 Laboratório de Produtos Bioativos, Instituto de Ciências Farmacêuticas, Universidade Federal do Rio de Janeiro, Macaé, RJ, Brasil; 4 Laboratório de Citogenética e Citometria, Departamento de Biologia Geral, Centro de Ciências Biológicas e da Saúde, Universidade Federal de Viçosa, Viçosa, MG, Brasil

**Keywords:** Plant stem cell, Coffea canephora, Proliferation, Regenerative Medicine, Tissue repair

## Abstract

Plant cell cultures have become a promising production platform of bioactive compounds for biomedical and cosmetic uses in the last decades. However, the success so far has been limited. The study aimed to evaluate the effectiveness of this unique biotechnology process to obtain a bioactive stem cell extract of *Coffea canephora* (SCECC) with antioxidant, anti-inflammatory, and regenerative properties. Total phenolic and flavonoid contents were determined in the SCECC by spectrophotometry. The chemical composition of the extracts was characterized by mass spectrometry. Antioxidant activity was evaluated using the colorimetric methods of free radical scavenging 2,2'-azinobis-3-ethylbenzothiazoline-6-sulfonic acid (ABTS) and the ferric reducing ability of plasma (FRAP). The anti-inflammatory activity was determined in lipopolysaccharide-stimulated RAW 264.7 macrophages through the production of superoxide anion (O_2_^•-^), nitric oxide (NO), tumor necrosis factor-alpha (TNF-α), interleukin-6 (IL-6), and the activity of nuclear factor kappa B (NF-κB). Moreover, the ability of SCECC to stimulate the proliferation and migration of fibroblasts was assessed. Five compounds were tentatively identified, two flavonoids, two phenolic acids, and one sugar. High phenolic content and antioxidant activity were observed in the SCECC. SCECC promoted the proliferation and migration of fibroblasts and suppressed the pro-inflammatory mediators O_2_^•-^, NO, TNF-α, and IL-6 in a dose-dependent manner. Moreover, SCECC inhibited the NF-κB transcription factor. Therefore, we obtained evidence that the extract from *C. canephora* stem cells can be used as a natural agent against skin damage. Hence, it could be of interest in cosmetics for preventing skin aging.

## Introduction

In the last decades, extensive work has been performed regarding the application of *in vitro* plant and organ cell culture technology in numerous areas. Different strategies have been developed to assess biomass accumulation and increase the synthesis of secondary metabolites and products that are useful in the food, pharmaceutical, cosmetics, and nutraceutical industries (1-3).

The production of bioactive compounds or extracts via field cultivation of plants has innumerous disadvantages such as low yields, production time, and fluctuations in the concentrations due to geographical, seasonal, and environmental conditions (1,2). Therefore, in recent years, various strategies have been developed to bypass these problems, and enthusiasm has grown for *in vitro* plant cell culture technology as an attractive alternative for the production of secondary metabolites and bioactive extracts (1). This biotechnological process is capable of producing bioactive products under controlled conditions and independent of external environmental factors, ensuring a continuous supply of uniform quality and high yields (4).

Extracts of plant stem cells are produced from *in vitro* tissue cultures, and their use in dermatology is extremely broad and safe, as many of the active metabolites do not produce immune system reactions (5). The compounds produced by plant stem cells are responsible for several of the positive effects of cosmeceuticals (6). The most well-known product based on plant stem cells launched recently was developed using the *Mallus domestica* and it showed anti-inflammatory, antioxidant, skin regenerative, and anti-aging activities, among other cellular protective activities (7). The activity of the plant stem cell culture extract of *Theobroma cacao* demonstrated great antioxidant capacity compared to conventional extracts from leaves and fruits (8). In addition, the antioxidant and metal-chelating activity of *Solanum lycopersicum* stem cell extract reflects the complexity of the active ingredients present in these extracts, suggesting that in addition to secondary metabolites, proteins, lipids, and carbohydrates with bioactive action are also present (6).

Plants are considered a rich source of bioactive products that can be used in the pharmaceutical and cosmetic industries (4). These phytochemical products can display antioxidant and anti-inflammatory activity by scavenging reactive oxygen species (ROS) and contributing to oxidative stress reduction. ROS, including superoxide radical (O_2_^•-^) and nitric oxide (NO), are mainly responsible for oxidative damage to cells (9). During oxidative stress, damage is caused in proteins, DNA, and cell membranes, triggering the signaling of the inflammatory process via nuclear factor kappa B (NF-κB). This transcription factor leads to the synthesis of inflammatory cytokines, such as tumor necrosis factor alpha (TNF-α) and interleukin (IL-6), which will lead to an even more intense degradation of the extracellular matrix (10,11). In this manner, fibroblasts reduce their proliferative cell cycle activity, reducing the synthesis of the extracellular matrix protein, and becoming a senescent cell (12).

Intrinsic skin aging occurs naturally and chronologically. However, aging is aggravated by extrinsic factors such as ultraviolet radiation (UV) from sunlight (13). The damage caused by the action of UV radiation promotes changes in the epidermis, increasing the thickness of the stratum corneum and thinning the epidermal layer, and even reducing the folds of the dermo-epidermal junction (14). In the dermis, there is a decrease in the content of collagen, glycosaminoglycans, and elastin. The decrease in the number of blood vessels is considerable (15).

*Coffea canephora* Pierre ex A. Froehner, a species of coffee, was selected for the present study. Coffee is one of the most important commodities worldwide and has a great economic impact in many countries. It is rich in polyphenols and phenolic compounds, such as chlorogenic acid, caffeic acid, and caffeine (16). These and other bioactive compounds have been reported to have high anti-inflammatory and antioxidant capacity. Hydrophilic extracts of *C. canephora* green seeds are promising sources of antioxidant compounds for the pharmaceutical and cosmetics industries (17). Thus, plant extracts of *Coffea* sp. are used in cosmetic products for combating skin aging and promoting rejuvenation. Recently, a liposomal formulation containing stem cell extract from *C. canephora* leaves was demonstrated to accelerate the wound healing process by increasing extracellular matrix formation and neovascularization and modulating the inflammatory response (18). For the first time, this study aimed to characterize the chemical composition and to investigate the antioxidant, anti-inflammatory, and fibroblast-stimulating activity of *C. canephora* stem cell extract *in vitro*. The mechanism of action of the potential anti-aging properties on the skin was also studied.

## Material and methods

### Cell lines

Fibroblast (L929) cell line (ATCC^®^-CCl1), macrophages (RAW 264.7) (ATCC^®^ TIB-71), and human embryonic renal cells (HEK-293) transfected with a luciferase-expressing gene (ATCC^®^ CRL-1573) were obtained from Cell Line Service, Brazil. The cells were cultured with DMEM (Dulbecco's modified Eagle's medium) supplemented with 10% fetal bovine serum, 100 IU/mL penicillin, and 100 μg/mL streptomycin, and maintained at 37°C in 5% CO_2._

### Cell aggregate suspensions from *C. canephora* leaves

**Figure 1:**
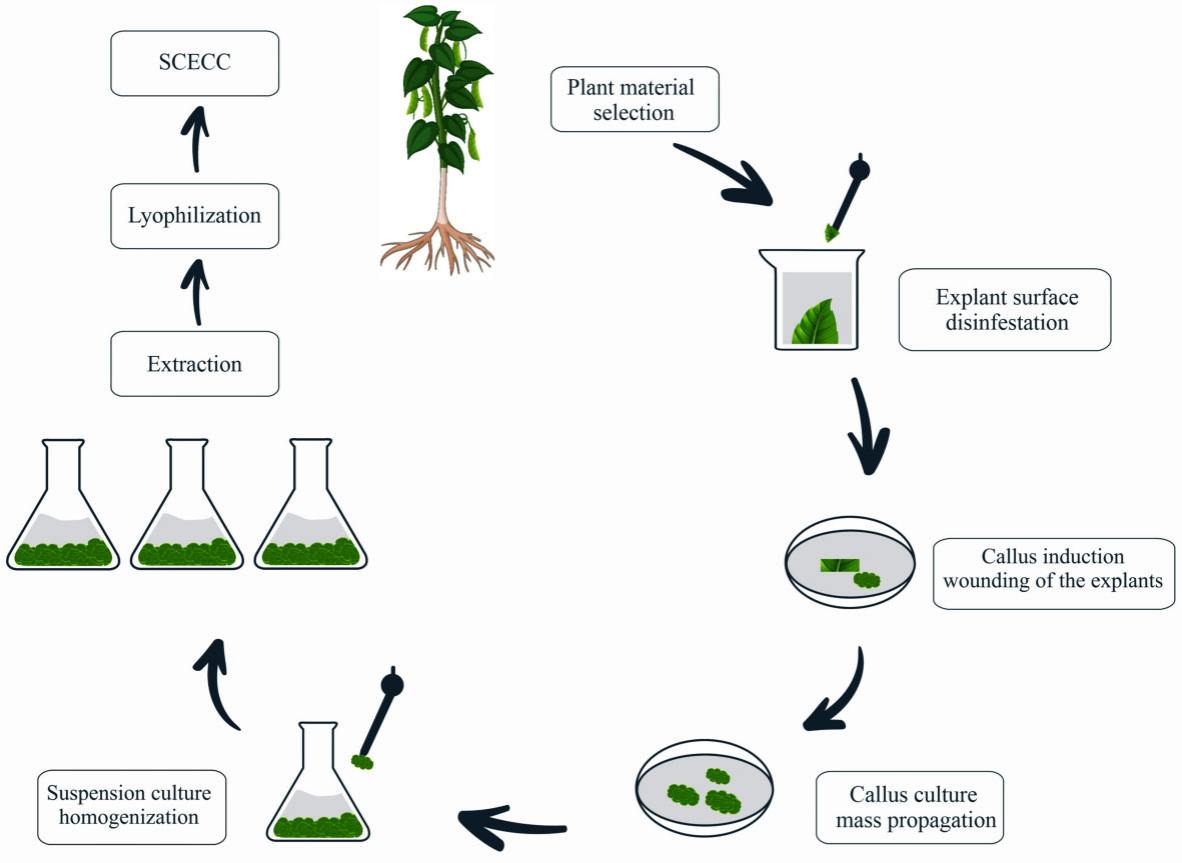
Guideline for the preparation of cell aggregate suspensions from *Coffea canephora* leaves. The leaf surface was disinfested and inoculated in a semisolid culture medium. The developed friable callus was propagated in a liquid medium, resulting in cell aggregate suspension. The stem cell extract of *C. canephora* (SCECC) was obtained from the stem cells of the aggregates in the culture medium after extraction and lyophilization.

*C. canephora* leaves were harvested from 10 individuals cultivated at the Espírito Santos Institute for Research, Technical Assistance and Rural Extension (INCAPER), Brazil (latitude 19°23′56-S, longitude 40°32′07-O). Leaves were washed with sterile H_2_O inside a hood, immersed in 70% ethanol, and subsequently in 3.5% NaOCl solution for surface disinfestation. Leaves were then washed 3 times in sterile H_2_O. Leaves explants were inoculated in Petri dishes containing semisolid friable callus induction medium and incubated at 24°C in the dark for 30 days (19). A total of 0.5 g of the resulting friable callus was transferred to Erlenmeyer flasks containing 30 mL liquid culture medium for establishment and multiplication of cell aggregate suspensions (19). The Erlenmeyer flasks were shaken at 110 rpm in the dark at 24°C. The cultures were sub-cultivated every 14 days for 2 months to increase biomass. In the end, cell aggregates in suspension and culture medium were processed using an ultrasonic probe followed by lyophilization. For extraction, the lyophilization was resuspended in ultrapure water (10% w/v), homogenized, and centrifuged at 3684 *g* for 15 min at 4°C. The obtained supernatant (extract) was named stem cell extract from *C. canephora* (SCECC, Figure 1).

### Antioxidant activity

The total antioxidant capacity of SCECC was determined by the ferric reducing/antioxidant power assay (FRAP) (20) and the radical scavenging activity of ABTS (2,2′-azinobis-3-ethylbenzthiazoline-6-sulfonate) (21). The results are reported as half maximal inhibitory concentration (IC_50_) (μg/mL).

### Total flavonoids and phenolic content

Total flavonoids were determined by the spectrophotometric method after a reaction with aluminum chloride (22,23). The values were calculated from a calibration curve using quercetin as standard and reported as mg of quercetin equivalent (QE) per 100 mL of SCECC. The total phenolic content was estimated by the spectrophotometric Folin-Ciocalteu method (22,23). The values are reported in grams of gallic acid equivalents (GAE)/100 mL SCECC as means±SD. Tests were performed in triplicate.

### Mass spectrometry analysis

Mass spectra were acquired using an LCQ Fleet Ion Trap ThermoFisher Scientific mass spectrometer (USA) as previously described with modifications (23). Sample introduction by direct infusion was performed by an MS syringe pump and an atmospheric pressure chemical ionization (APCI) source (ThermoFisher Scientific) operating in positive- or negative-ion modes. MS was operated using the following parameters: capillary voltage, 3.6 kV; drying temperature, 350°C; vaporizer temperature, 400°C; nebulization, 45 psi; drying gas flow 7 L/min; corona current, 4000 nA; and collision energy, 35 eV. Samples were prepared at a concentration of 1 mg/mL using acetonitrile:ultrapure water (1:1, v/v) as solvent. Acetonitrile of chromatographic grade was purchased from Tedia (USA). Water was purified using a Milli-Q Plus system (Millipore, USA). Signals were acquired in m/z range from 100 to 1000 and for MS2 of the base peaks. The results were compared with the literature for the compounds tentatively identified.

### *In vitro* cytotoxicity

Cell viability was assessed using the colorimetric MTT test (24). Briefly, RAW 264.7 macrophages and L929 fibroblasts were seeded in 96-well plates at 8×10^4^ cells/well. After overnight, cells were treated with different SCECC concentrations (31.2 to 1000.0 μg/mL) for 24 h at 37°C in 5% CO_2_. Then, the MTT solution was added to each well and the formazan crystals were dissolved with dimethyl sulfoxide (DMSO). The absorbance of each well was measured at 595 nm wavelength by a microplate reader (Multi-Mode Microplate Reader, Filter Max F5, Molecular Devices, USA). The results are reported as a percentage of viable cells.

### Nitric oxide analysis

RAW 264.7 macrophages were plated at a concentration of 8×10^4^ cells/mL in 96-well plates and incubated overnight (37°C at 5% CO_2_). SCECC was added at concentrations 1-100 μg/mL. Lipopolysaccharide (LPS) (1 μg/mL) was added to all wells after 1 h, except for the negative control. L-NIL (30 μM) was used as a positive control. After 20 h of incubation, 100 μL of the supernatant was transferred to another plate and the nitrite quantification was performed by adding 100 μL of Griess solution (25,26). Results are reported as means±SD of the nitrite concentration (μM) calculated by regression analysis of a standard curve of sodium nitrite.

### Superoxide anion assay

The superoxide assay was used to determine the inhibitory effect of SCECC on the production of the superoxide radical (O_2_^•-^) in LPS-stimulated RAW 264.7 macrophages, as previously described by Soares et al. (23). The results are reported as means±SD of superoxide anion production (%).

### Measurement of cytokines

The supernatant of LPS-stimulated macrophages cell culture exposed to different concentrations of the SCECC extracts (1-100 μgmL) was used to quantify IL-6 and TNF-α by enzyme-linked immunosorbent assay (ELISA), according to the manufacturer's instructions (eBioscience, USA).

### NF-κB activity

The NF-κB inhibition assay was performed using a human renal cell line transfected with NK-κB-luciferase gene reporter as previously described in the literature (26,27) and following the manufacturer's instructions (Promega, USA).

### *In vitro* cell migration assay - scratch assay

The experimental model used to evaluate fibroblast migration stimulated by SCECC was the scratch assay (28). Briefly, fibroblasts were cultured in 6-well plates until they reached a monolayer confluence. A linear artificial scratch was induced in the monolayer with a sterile 100-μL tip. Then, the monolayers were incubated for 14 h with different concentrations of SCECC (10-100 μg/mL). Platelet-derived growth factor (PDGF) was used as a positive control. After the cells were fixed and stained with DAPI (2-(4-amidinophenyl)-1-indole-6-carboxamidine), the total cell number in the wounded area was quantified using CellC^®^ software (http://en.bio-soft.net/draw/CellC.html). Results are reported as a percentage of cells that migrated and/or proliferated into the injured area compared to the untreated control group.

### BrdU proliferation assay

The effects of SCECC on fibroblast proliferation were measured with the thymidine analog BrdU (5-bromo-2'-deoxyuridine), following its incorporation into newly synthesized DNA and its subsequent detection with an anti-BrdU antibody according to the instructions and specification of the manufacturer (Roche^®^, Germany).

### Statistical analysis

Statistical analyses were performed using GraphPad Prism 8 software (USA). Data are reported as means±SD. Statistical comparisons were carried out using univariate analysis of variance (ANOVA) followed by Tukey's *post hoc*. Values of P<0.05 were considered significant. All experiments were carried out at least in triplicate.

## Results

### Cell aggregate suspensions from *C. canephora* leaves

From the *C. canephora* leaves, friable calli were obtained in a semisolid medium. The friable calli showed pale-yellow color, and the cell mass progressively increased over time in a semisolid medium, allowing the cell aggregate suspension establishment. As well as the friable calli in a semisolid medium, the cell mass of the *C. canephora* aggregates increased in the liquid medium during the subcultures. Thus, we obtained the SCECC for further analysis.

### Antioxidant properties

The SCECC had a high ABTS radical scavenging, with IC_50_ of 58.15±12.4 μg/mL, while the IC_50_ of chlorogenic acid was 6.32±0.21 μg/mL. In addition, FRAP exhibited an IC_50_ of 15.52±0.21 μg/mL and the chlorogenic acid presented an IC_50_ of 4.71±0.17 μg/mL (Table 1).

**Table 1:** *In vitro* antioxidant activity of stem cell extracts of *Coffea canephora* (SCECC) by ABTS free-radical scavenging activity and by ferric reducing antioxidant power (FRAP).

	Antioxidant activity (IC_50_ µg/mL)	
	ABTS	FRAP
SCECC	58.15±12.4^a^	15.52±0.21^a^
Chlorogenic acid	6.32±0.21^b^	4.71±0.17^b^

Data are reported as means±SD. Tests were performed in triplicate (n=3). Different letters in the same column indicate significant differences (P<0.05, one-way ANOVA followed by Tukey's *post hoc* test). IC_50_: half maximal inhibitory concentration.

### Total flavonoids and phenolic content

SCECC presented a lower content of flavonoids compared to phenolic compounds. The flavonoids content was 7.65±0.05 mg of quercetin equivalent (QE) per 100 mL of SCECC, while the total phenolic compounds were 974.12±0.06 mg of gallic acid equivalents (GAE) per 100 mL SCECC, indicating a high content of phenolic compounds.

### Chemical composition of SCECC

**Figure 2:**
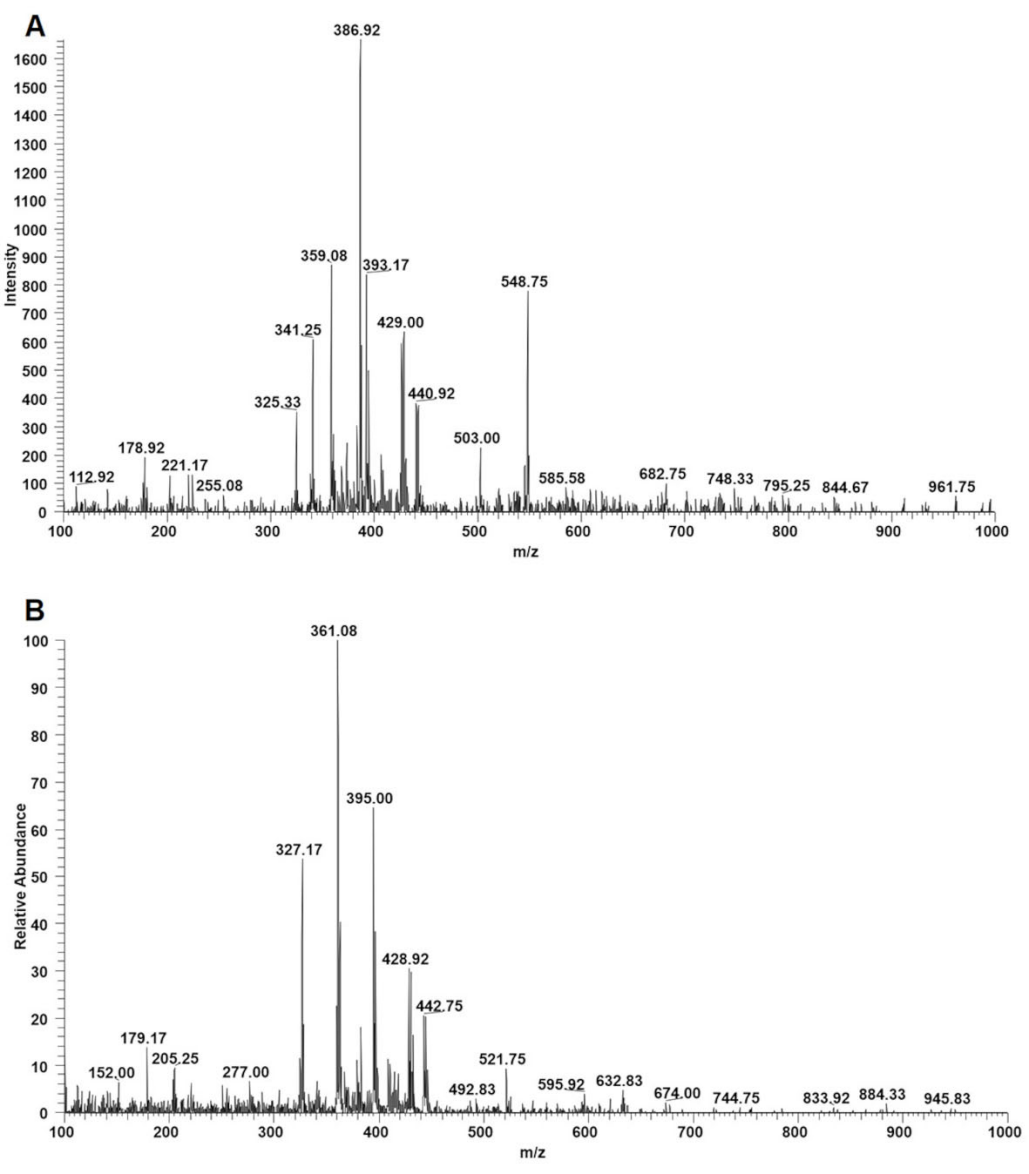
Representative APCI-mass spectrometry for the stem cell extracts of *Coffea canephora* in (**A**) negative and (**B**) positive ionization.

The SCECC was analyzed in both negative (Figure 2A) and positive (Figure 2B) APCI ionization modes with monitoring mass range from 100 to 1000 m/z. The processing of extracts and comparison of spectra obtained by MS-MS of extracts in the literature led to the preliminary identification of five compounds (Table 2; refs 29−31), using negative ionization mode, suggesting two flavonoids, two phenolic acids, and one sugar. The identified flavonoids were rutin and catechin gallate. The catechin gallate presented an m/z of 441 (M−H)^−^ for the deprotonated molecule and fragment ion at m/z 289 (M−H−152)^−^, suggesting the presence of a galloyl unit. In turn, rutin presented an m/z of 609 (M−H)^−^ and the fragment ion at m/z 301 indicated the aglycone quercetin deprotonated molecule.

**Table 2:** Compounds tentatively identified in the stem cell extracts of *Coffea canephora*.

Compound	[M−H]^-^	Fragment ions	Reference
Caffeic acid	179	161, 135	(29)
Sucrose	341	179, 161	(29)
Rosmarinic acid	359	197, 179, 161	(30)
Catechin gallate	441	289	(31)
Rutin	609	301	(29)

[M−H]^−^: deprotonated molecule.

The preliminary identified phenolic acids were caffeic and rosmarinic acids. The caffeic acid revealed an m/z of 179 (M−H)^−^ and the most commonly observed fragment at m/z 135 was related to CO_2_ (44 Da) neutral loss due to the α elimination mechanism, which is a typical charge migration fragmentation in deprotonated compounds. Rosmarinic acid presented an m/z of 359 (M−H)^−^, and the main fragments are m/z=197, the deprotonated 3,4-dihydroxy phenyllactic acid, and m/z*=*179 for the deprotonated molecule of caffeic acid (32). Moreover, also tentatively identified, the disaccharide sucrose of m/z 341 (M−H)^−^ presented as main fragments at m/z 179 and 161, both generated from glycosidic bond cleavage.

### MTT cytotoxicity

SCECC did not exhibit any cytotoxic effects against the non-cancerous L929 fibroblasts and murine macrophage RAW 267.7 cells up to 1000 μg/mL.

### Nitric oxide and superoxide anion analysis

**Figure 3:**
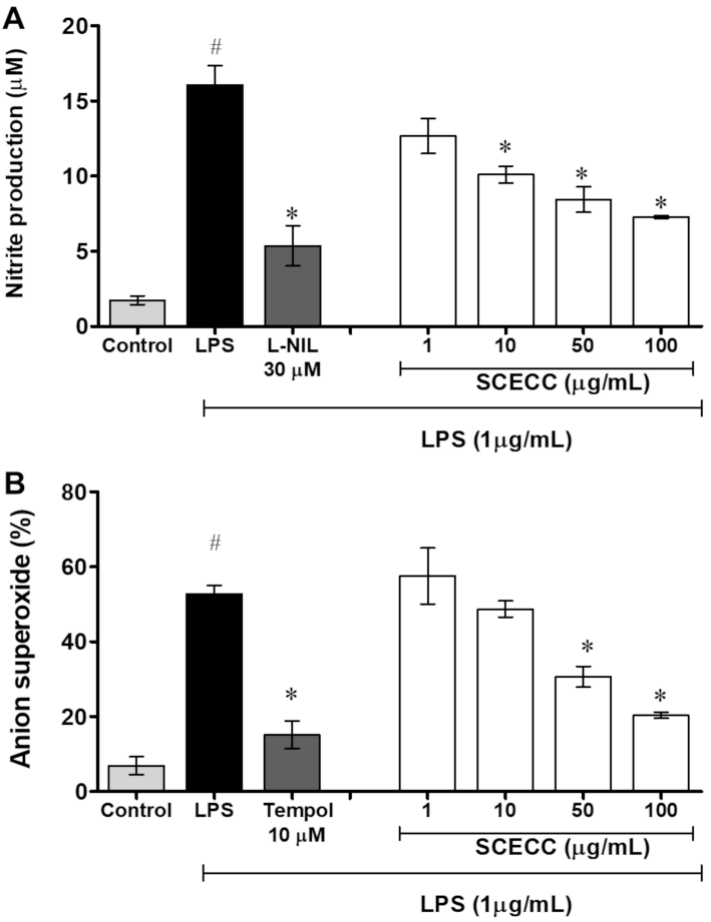
Effect of the stem cell extracts of *Coffea canephora* (SCECC) on the production of (**A**) nitric oxide (NO) and (**B**) superoxide radical (O_2_^•-^) production. RAW 264.7 macrophages were exposed to different SCECC concentrations and stimulated with lipopolysaccharide (LPS) (1 μg/mL). L-NIL 30 μM and Tempol 10 μM were used as positive controls. The data are reported as means±SD. ^#^P<0.05 compared to the basal control group; *P<0.05 compared to LPS-induced cells (one-way ANOVA followed by Tukey's *post hoc* test).

The SCECC significantly decreased the production of inducible nitric oxide in a dose-dependent manner in LPS-induced RAW 267.2 macrophages (Figure 3A). Significant reductions of 37.1±1.1%, 47.4±0.9%, and 54.7±1.6% were observed after treatment with 10, 50, and 100 μg/mL SCECC, respectively. SCECC also showed antioxidant action against superoxide anion (O_2_^•-^). The effects were dose-dependent compared to the LPS control group. Significant reductions of 41.9±2.1% and 61.4±1.9% were observed after treatment with 50 and 100 μg/mL SCECC, respectively (Figure 3B).

### Inhibition of cytokine production

**Figure 4:**
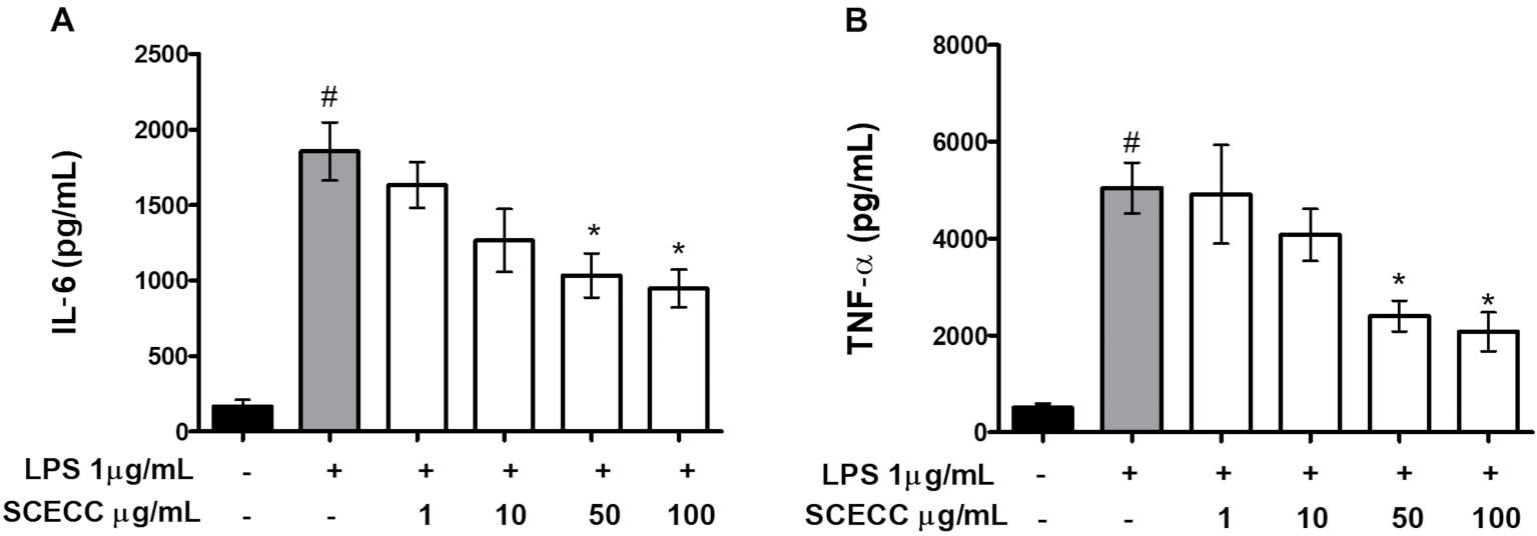
Effect of stem cell extracts of *Coffea canephora* (SCECC) on the concentration of pro-inflammatory cytokines interleukin (IL)-6 (**A**) and tumor necrosis factor-alpha (TNF-α) (**B**). RAW 264.7 macrophages were exposed to different SCECC concentrations in the presence or absence of lipopolysaccharide (LPS). Data are reported as means±SD. ^#^P<0.05 compared to the basal control group; *P<0.05 compared to LPS-induced cells (one-way ANOVA followed by Tukey's *post hoc* test).

After 24 h of LPS stimulation of macrophages, the release of IL-6 and TNF- α significantly increased in the cellular supernatant, indicating an inflammatory macrophage response. In contrast, the SCECC significantly reduced the production of IL-6 and TNF-α at 50 and 100 μg/mL (Figure 4).

### Inhibition of NF-kB activity

**Figure 5:**
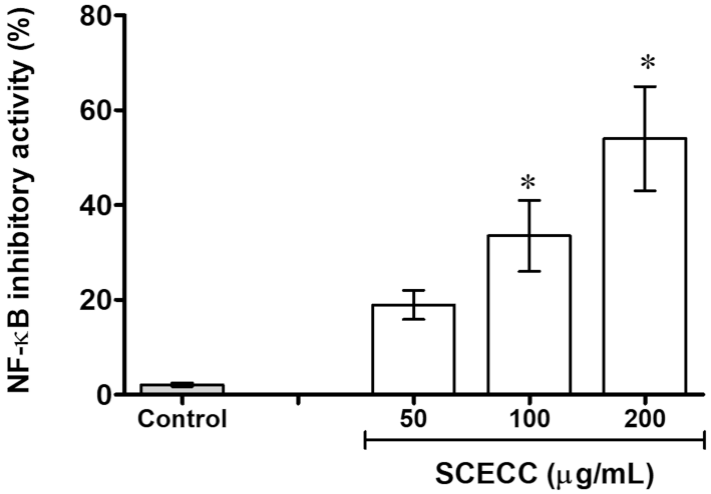
Effect of the stem cell extracts of *Coffea canephora* (SCECC) on the inhibitory activity nuclear factor kappa B (NF-κB). HEK 293 cells were exposed to different SCECC concentrations and incubated for 6 h with or without TNF-α (2 ng/mL). Data are reported as means±SD of the percentage of NF-κB inhibitory activity. *P<0.05 compared to the control (one-way ANOVA followed by Tukey's *post hoc* test).

SCECC inhibited the NF-κB activity of about 31.4±6.4% and 51.9±10.7% after treatment with 100 and 200 μg/mL, respectively (Figure 5).

### *In vitro* cell proliferation and migration activity

**Figure 6:**
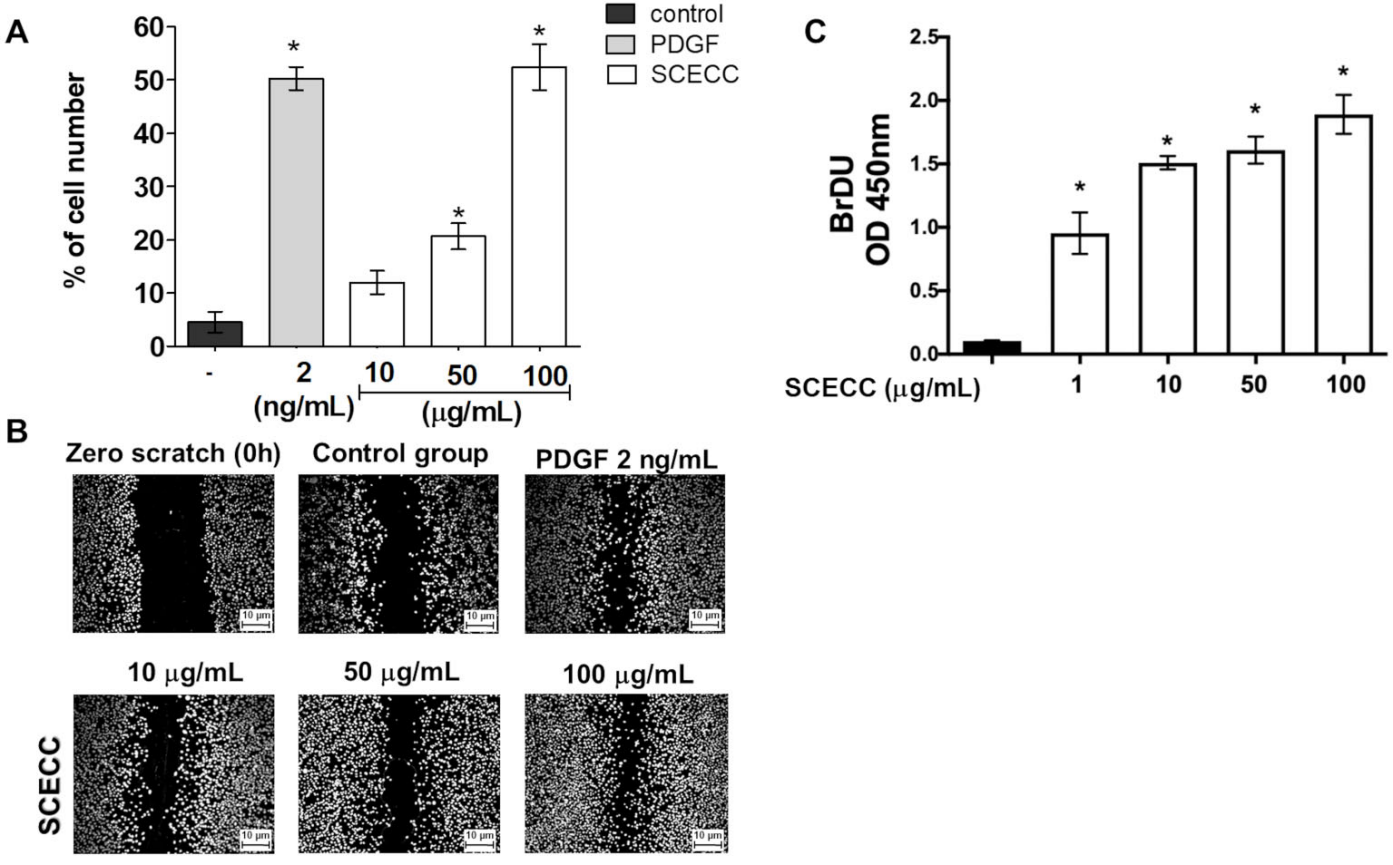
Influence of stem cell extracts of *Coffea canephora* (SCECC) on fibroblast proliferation and migration. **A**, Percentage of total cell number in the scratch area after 14 h of incubation (37°C at 5% CO_2_) without (control) or with SCECC at 10, 50, and 100 µg/mL and PDGF at 2 ng/mL (positive control). Data are reported as means±SD of three independent experiments. ^#^P<0.05 compared to untreated cells (control); *P<0.05 compared to PDGF (one-way ANOVA followed by Tukey's *post hoc* test). **B**, Representative microscope images of the cellular wound immediately after injury (zero scratch 0 h) and 14 h post-treatment, as indicated in each panel. **C**, Effect of the SCECC on fibroblast proliferation. Data are reported as as means±SD of the absorbance on 450 nm wavelength. *P<0.05 compared to untreated cells (control) (one-way ANOVA followed by Tukey's *post hoc* test).

Our results showed that SCECC promoted the migration of fibroblasts dose-dependently at 50 and 100 μg/mL (Figure 6A and B). In addition, specifically at 100 μg/mL, the result was similar to the PDGF-positive control. SCECC-treated cells also showed a significant increase in BrdU incorporation at DNA, indicating a stimulatory effect on fibroblast proliferation measured by the absorbance of the culture in all tested concentrations (Figure 6C).

## Discussion

For the first time, the effectiveness of this unique biotechnology process for obtaining a bioactive stem cell extract of *Coffea canephora* (SCECC) was evaluated and its pharmacological properties were demonstrated as a natural extract for improving skin health, tissue repair, prevention, and treatment of skin aging.

Anti-aging properties, mainly due to antioxidant activities, have already been described in many extracts from stem cells acquired from *in vitro* culture (3). This study demonstrated the antioxidant, anti-inflammatory, and skin regenerative potential of SCECC. The flavonoids and phenolic compounds present in SCECC exhibited antioxidant and anti-inflammatory properties. In addition, the SCECC stimulated fibroblasts, increasing their proliferative capacity (cell cycle activation), as well as migration. Generally, oxidative stress can be reduced by antioxidants present in food or applied topically to the skin (33). SCECC showed antioxidant action by decreasing the production of O_2_^•-^ and NO^•^ radical release, both inflammatory signals. This action was attributed in part to the presence of phenolic compounds and flavonoids. Plants have efficient enzymatic and non-enzymatic antioxidant defense systems to avoid the toxic effects of free radicals. Thus, in plant stem cell cultures, such antioxidant efficiency may be high due to the complexity of secondary metabolites produced during culture proliferation (5,34).

Skin not exposed to sunlight only undergoes intrinsic aging. Photoaging is the sum of natural aging of the skin associated with the action of ultraviolet radiation. In both cases, aging can lead to a decrease in the structural integrity of the skin and loss of physiological function, especially of the extracellular matrix (9). Ultraviolet radiation can cause an increase in ROS, damage the structure and function of cells, and mediate inflammatory responses. ROS induce and accelerate the aging process, including skin aging, by mediating inflammation through activation of AP-1 and suppression of TGF-β, activating the NF-κB signaling pathway, which results in increased release of matrix metalloproteinases (MMP) and decrease in collagen, elastin, and extracellular matrix synthesis (35).

SCECC inhibited the NF-κB signaling pathway. This result is important to predict the possible anti-inflammatory and antiaging mechanism of the SCECC action. Similarly, SCECC significantly reduced the concentrations of pro-inflammatory cytokines IL-6 and TNF-α in LPS-stimulated macrophage cultures. Reactive oxygen and nitrogen species can act as modulators of transcription factors, including NF-κB. This transcription factor regulates multiple aspects of adaptive and innate immune system cells and plays a pivotal role in the inflammatory process. NF-κB induces the transcription of pro-inflammatory mediators like inducible NO synthase, TNF-α, IL-1, and IL-6 (27). Therefore, the inflammatory process involves the release in a cascade of pro-inflammatory and anti-inflammatory cytokines and plays a major role in the initiation of local and systemic inflammatory processes (27). Taking this into account, the modulation of the inflammatory process is considered an important step to reduce extracellular matrix degradation and preserve protein structures (11).

Recently, a liposome cream obtained from the stem cell of *C. canephora* modulated the inflammatory process and increased the extracellular matrix formation, acting as a tissue regenerator and stimulator of collagen production and skin neovascularization (18). In addition, the liposome cream decreased pro-inflammatory cytokines such as TNF-α and IL-6 and increased IL-10 (18). The presence of secondary metabolites in SCECC defines a large part of its biological activity, but this extract is more complex, as it derives directly from a lyophilized culture medium where proteins, lipids, carbohydrates, and plant cytokines are present. Part of the activity observed in the present study was due to this complexity, as shown by Tito et al. (6), who demonstrated the metal-chelating activity of *S. lycopersicum* stem cells attributed to the presence of complexing proteins in the plant extract. In addition, *S. lycopersicum* stem cell extract is rich in flavonoids and phenolic acids and has high antioxidant activity, protecting skin cells from oxidative stress (6). Wu and Zhong (36) demonstrated an increase in the biological activity of *Panax ginseng* through the production of plant culture extracts, highlighting the activity of saponins and polysaccharides. In addition, the extracts from *Calendula officinalis* flowers stimulated the proliferation and migration of fibroblasts in an *in vitro* wound healing assay, demonstrating the potential of plant extracts in cell migration and proliferation (28). This effect is important considering cellular senescence, where the fibroblast gradually and progressively decreases its metabolic activity due to chronological aging, which can be aggravated by oxidative stress and inflammation (37). SCECC seemed to promote the necessary stimulus for the fibroblast to maintain its full functional and proliferative capacity (38). As verified here for SCECC, *Coffea bengalensis* stem cell extract was able to induce collagen synthesis in fibroblasts and reduce intracellular ROS, indicating that it protects skin cells from free radical damage (39). In this context, we report for the first time that SCECC also contributes significantly to fibroblast proliferation and migration, making it a promising target in the search for new natural cosmetics.

**Figure 7:**
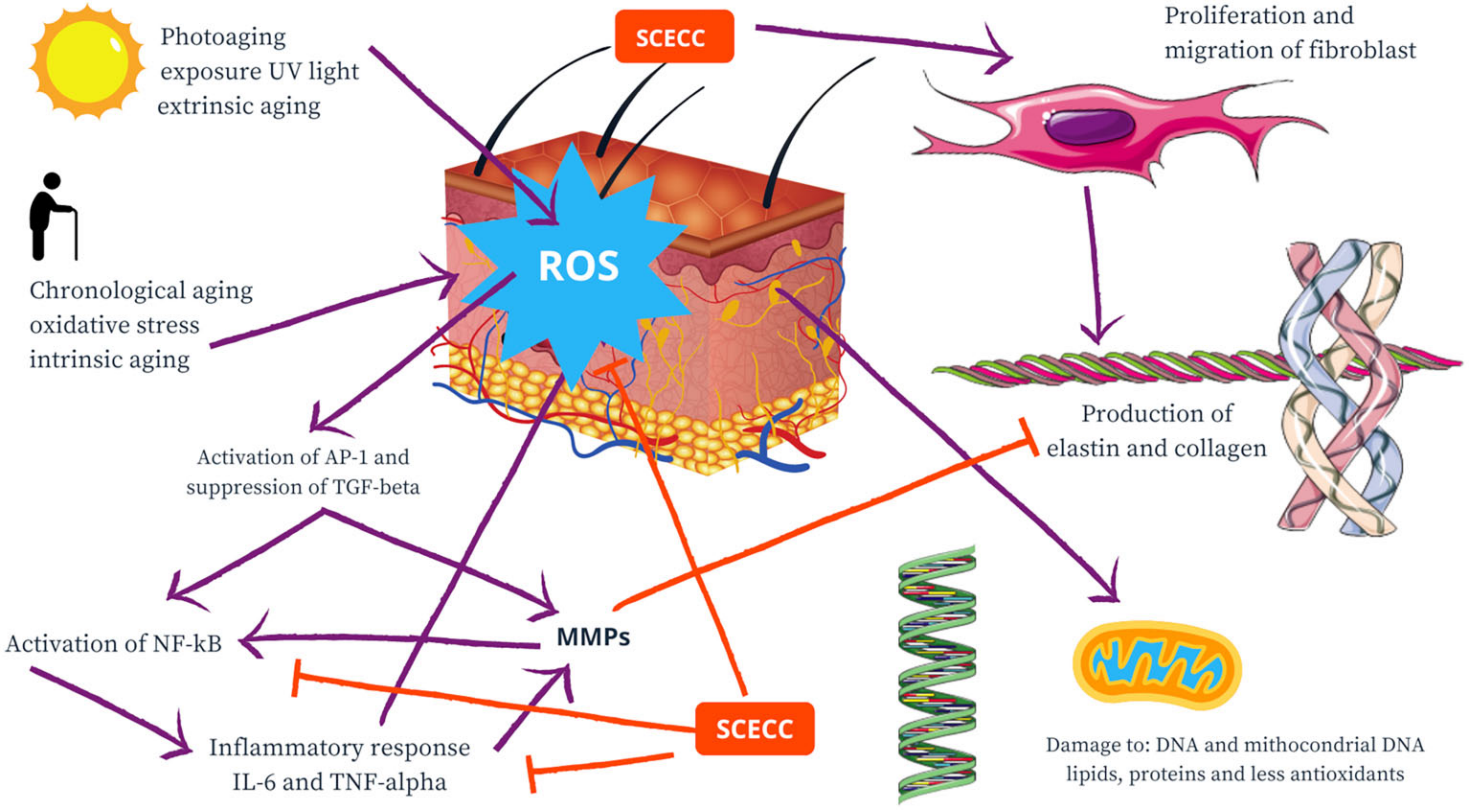
Schematic representation of the main results obtained with the stem cell extract of *Coffea canephora* (SCECC).

In summary, SCECC had antioxidant properties as it decreased the formation of free radicals that promote oxidative stress. SCECC can decrease the production of NF-κB, a transcription factor of inflammatory proteins, and decrease the action and release of inflammatory IL-6 and TNF-α. SCECC has also a fibroblast-stimulating action, increasing protein synthesis and proliferation of fibroblasts. In addition, phenolic compounds such as rosmarinic and caffeic acid and flavonoids such as rutin were detected in SCECC, which may contribute to its biological properties (Figure 7).

Every research is conducted with a purpose. Although this study brought many insights and perspectives about the potential of biotechnological processes to obtain bioactive compounds for biomedical and cosmetic uses, some limitations can be addressed. While the results are promising, it is important to verify the effectiveness of SCECC *in vivo*, particularly in animal models, to ensure its safety and efficacy. The study only focused on the bioactive properties of SCECC, particularly its antioxidant, anti-inflammatory, and regenerative properties. Other properties, such as its toxicity, stability, and shelf-life, should also be investigated before it can be considered for commercial use.

This study successfully produced, for the first time, a stem cell extract of *C. canephora* and showed its pharmacotherapeutic potential, which perhaps could be incorporated into bioproducts for skin rejuvenation and aging prevention.
